# Advantageous effects of bentonite on growth performance and metabolic compounds of two mesophytic plants in desert sandy soils

**DOI:** 10.1186/s12870-025-07834-7

**Published:** 2025-12-19

**Authors:** Farghali K.A., Suzan A. Tammam

**Affiliations:** Department of Botany and Microbiology, Faculty of Science, Assiut University, Assiut, 71,516 Egypt

**Keywords:** Bentonite, Desert sands, *Senna*, *Zea*, Growth, Metabolites

## Abstract

**Background:**

The best use of sandy soil in hot arid and semi-arid lands for cultivation is the ability to improve its physical and chemical properties. Therefore, the application of eco-friendly and natural substances added to sandy soils has been proven to improve water retention and alleviate nutrient deficiencies, consequently enhancing agricultural outputs. The present study aimed to investigate the advantageous effects of applying natural substances, particularly bentonite (a Type of clay), to improve the quality of desert sandy soils, and attributes of two mesophytes (cultivated *Zea mays* and wild *Senna occidentalis*) growing in three sandy soils were studied.

**Results:**

The data obtained indicated that the morphological parameters, such as length and fresh weight of shoot and root in *S. occidentalis* and *Z. mays* plants, were positively affected by the soil texture, bentonite, and their interaction. In calcareous sandy soil, the water content of soil, shoot, and root tended to a maximum value with added a certain concentration of bentonite was added. With fine sands, the total chlorophyll content in both plants was markedly increased with the application of bentonite, which had a dominant role. The same role of bentonite was an effect on the different metabolic compounds, such as total soluble proteins, free amino acids, and total soluble sugars in the organs of the plants investigated. Fine sand exerted a high content of soluble proteins and soluble sugars, whereas the calcareous sandy soil accelerated the free amino acids in various plant organs. In general, bentonite had a major role in the osmo-metabolic compounds of both plant organs, as well as in the soluble proteins of *Senna occidentalis* root. Significant correlations existed between the water content of soil and plant organs with morphological and metabolic compounds.

**Conclusion:**

This investigation studies the advantageous effects of bentonite to improve the quality of desert sandy soil and plant productivity. Bentonite had a positive effect on the growth and metabolites of *Zea mays* and *Senna occidentalis* growing in three sandy soils. Also, bentonite affects the chlorophyll and different metabolic compounds (total soluble proteins, free amino acids, and soluble sugars) in two plants.

## Introduction

Plant growth and development are mainly based on the soil quality, water availability and nutrients acquired by plant roots. In arid and semi-arid climates, sandy soils have severe consequences for plantations. Therefore, the improvement of sandy soil can be achieved by using natural amendments such as bentonite. Hence, the addition of bentonite may be an effective approach for solving many problems affecting biochemical activity and plant growth under harsh environmental conditions [1].

The reclamation of sandy soil through the application of bentonite has beneficial effects on water retention and nutrient availability. Also, bentonite addition as a colloidal substance has a capacity to mitigate the nutrient ion leaching by binding on its surface [2, 3] and has been proven to improve soil water retention and nutrients necessary for plant productivity [4, 5]. Furthermore, the bentonite exhibited a synergistic effect for the growth of plants in sandy soils [6]. The addition of these amendments has the potential for improving plant photosynthesis, ultimately photosynthetic capacity, and plant carbon metabolism [7]. Thus, photosynthetic rate exhibited a high value, attributed to increase chlorophyll content in the plant leaf. Bandian et.al [8] revealed that the high chlorophyll content was found in beds possessing high bentonite levels. Additionally, the essential element contents in bentonite could increase plant biomass and make more exchange sites available to hold plant nutrients for plant growth [9]. Moreover, increasing nutrients in bentonite increased the root ability to uptake more elements such as P and N consequently, increasing in different plant metabolites [10]. Furthermore, the accumulation of K in roots with bentonite application can increase the ions related to osmolality. Consequently, the accumulation of metabolites and ions in plants serves as compatible solutes, which are related to osmotic adjustments.

In general, sandy soil is poor in retained water and weakness in its chemical properties leads to free percolation because of a deficiency of fine particles. Therefore, the goal of this investigation was an attempt to understand the role of bentonite as a natural source for improving the sandy soil inhabits warm desert which participates in retaining water and sufficient nutrients, leading to increased plant cropping and productivity. Accordingly, the effect of bentonite, soil texture, and their interaction on some growth and physiological parameters in two mesophytic plants (*Zea mays* L., cultivated plant and *Senna occidentalis* (L) Link, wild plant) was studied.

## Materials and methods of bentonite

Two plants, namely *Zea mays* L., var. *everta* Sturt. (cultivated plant) and *Senna occidentalis* (L.) Link (wild plant) were used for experimentation. Experimental seeds of *Z. mays* were supplied by the Crop Science Department, Faculty of Agriculture, Assiut University. *Senna occidentalis* is a wild mesophyte growing in the Botany farm of Assiut University and the specimens of the plant were deposited in Assiut University herbarium (ASTU), Egypt, after being identified by K.A. Farghali, professor of Plant Ecology, according to Tackhlom [11] and Boulus [12]. The seeds of *senna* were collected from the plant during the spring season. Plants were grown in plastic pots containing 400 g of air-dry soil, representing three types of soil textures that contribute to different ecological affiliations of the Egyptian desert. These soils were collected from 1- Mediterranean coastal dunes have oolitic calcareous sandy soil (Cal. soil), 2- Wadi El-Assiuti in the eastern desert have coarse sandy soil (C. soil) and Kharga Oasis in the western desert have fine sandy soil (F. soil). Each type of soil incubated seed containing certain amounts of bentonite levels as follows: 0% as control, 1.5%, 3% and 5% of the soil weight. Three pots (replicates) were assigned at random to each treatment level. Soil was irrigated with distilled water reaching field capacity.

The experiment was carried out under natural laboratory conditions, where the air temperature was 36–26 ^◦^C during the day and night, respectively, relative humidity of the air was 40–43% with daily artificial and natural illumination. Germination of seeds was carried out in the plastic pots and the emergence of plumule took place after 2 days in both *Zea mays* and *Senna occidentalis*. At the end of the experiment (two weeks for *Zea mays* and four weeks for *Senna occidentalis* due to deceleration of growth in *Senna* as a wild plant), the growth parameters of healthy seedlings of the two investigated plants were detected. These parameters included: shoot and root length, fresh weight of shoot and root, shoot/root ratio of length, shoot/root ratio of fresh weight, water content of the two plant organs and soil water content.

### Determination of total chlorophyll (Chl.) content

In fresh leaves, the total chlorophyll (*a* + *b*) content was extracted from fresh leaves using 85% acetone and quantified according to Lichtenthaler [13].

### Preparation of plant extracts for analysis

For the determination of water-soluble metabolites, the seedlings of both species were washed with distilled water and dried thoroughly with filter paper. Excised organs (shoots and roots) were freshly weighed (0.5 g) and homogenized in 10 ml of ice-cold distilled water, followed by centrifugation at 7000 rpm for 15 min. The supernatant was transferred to a glass bottle; the extracts were subjected to deep freezing until analysis.

### Determination of soluble nitrogen metabolites

Soluble proteins (S.P.) and free amino acids (A.A.) were determined according to procedures described by Lowry et al. [14], Lee and Takahanshi [15], respectively. The content of each metabolite in the different experimental plants has been expressed in mg/g. fresh wt. by using the spectrophotometer UNICAM model UV-Vi's spectrometry (Made in England).

### Determination of total soluble sugars (S.S)

In the two plants investigated, both organ (shoot or root) extracts, total soluble sugars were determined according to Dubois et al. [16].

### Statistical analysis

The effects of single factors (soil type and percentage of bentonite) and their interaction (Soil texture x bentonite) were evaluated via analysis of variance (F values), The share % is used to evaluate the relative role of each single factors and their interaction in the contributing to the total effect of treatment combinations, (the share % is extracted from the summation of square of each factor related to the total summation square of treatments). The sharing percentage and the simple linear correlation coefficient, r., was calculated according to Ostle. [17]. This statistical analysis was conducted using the SPSS program [18].

## Results

**Fig. 1:**
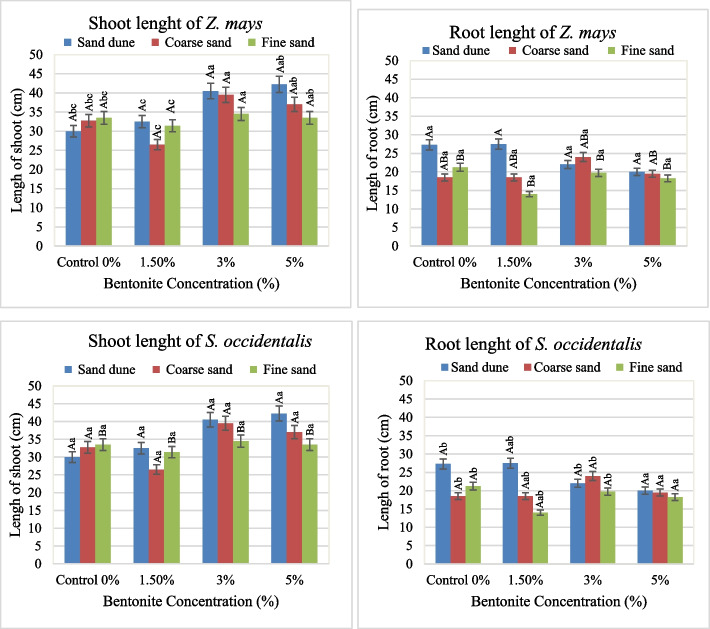
Average values of shoot and root length (cm) of plants investigated at different bentonite concentrations (%) and soil textures, [values having similar symbols (A, B. soil texture and a, b. bentonite conc.) indicate no significant difference according to the Tukey test]

**Fig. 2:**
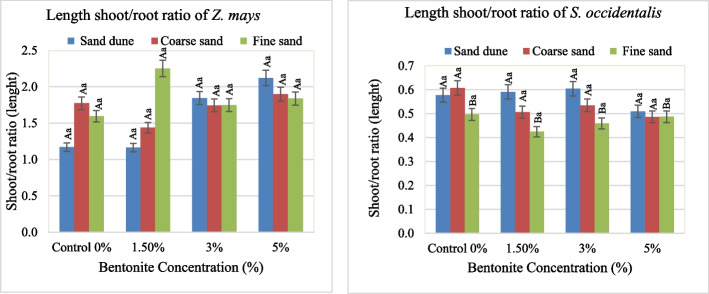
Average values of shoot/root ratio of length of plants investigated at different bentonite concentrations (%) and soil textures [values having similar symbols (A, B. soil texture and a, b. bentonite conc.) indicate no significant difference according to the Tukey test]

**Fig. 3:**
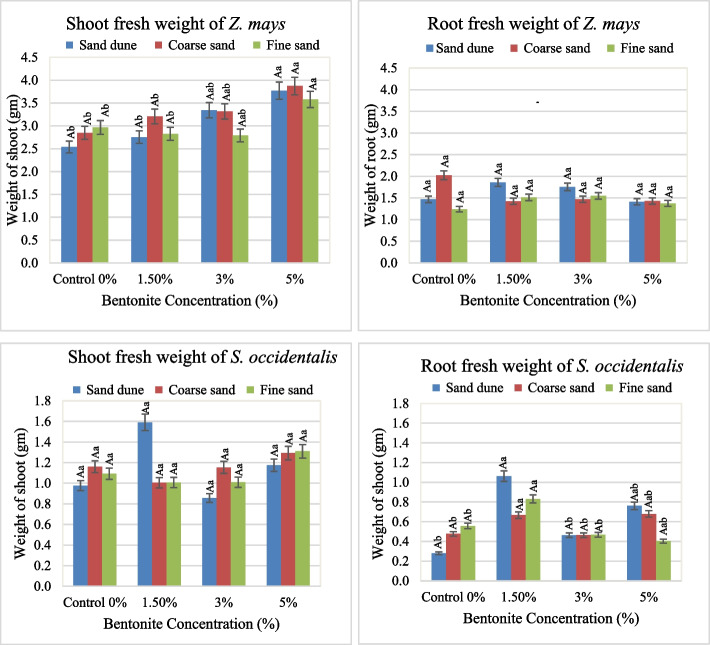
Average values of shoot and root fresh weight of plants investigated at different bentonite concentrations (%) and soil textures [values having similar symbols (A, B. soil texture and a, b. bentonite conc.) indicate no significant difference according to the Tukey test]

**Fig. 4:**
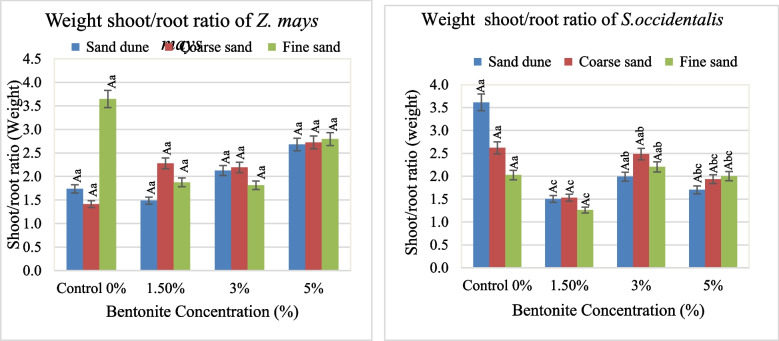
Average values of the weight shoot/root ratio of plants investigated at different bentonite concentrations (%) and soil textures [values having similar symbols (A, B soil texture and a, b bentonite conc.) indicate no significant difference according to the Tukey test]

**Fig. 5:**
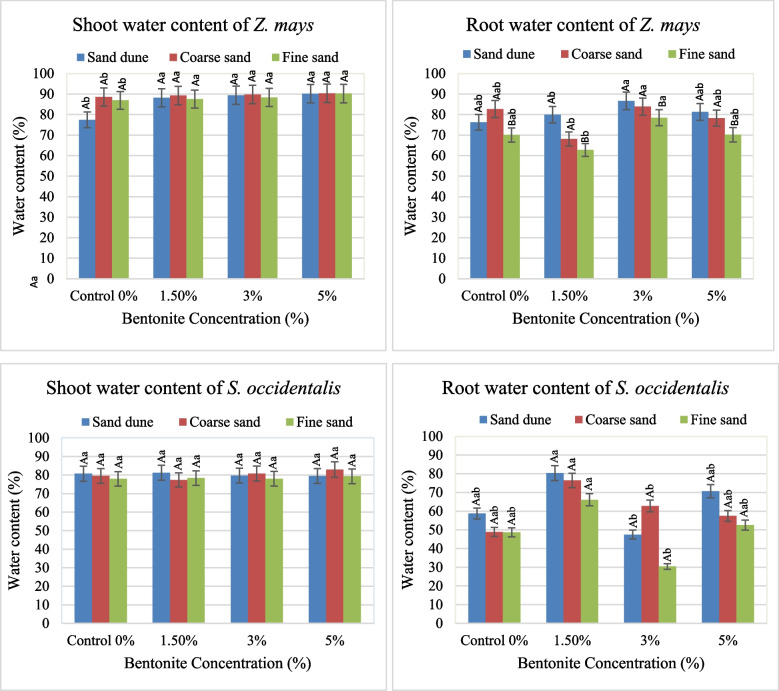
The average values of shoot & root water content of both plants investigated at different bentonite concentrations (%) soil texture, [values having similar symbols (A, B. soil texture and a, b. bentonite conc.) indicate no significant difference according to the Tukey test]

**Fig. 6:**
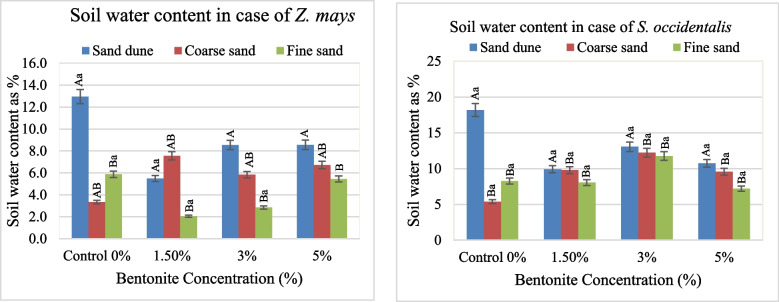
Average values of water content in the soil cultivated by plants investigated at different bentonite concentrations (%) and soil textures [values having similar symbols (A, B. soil texture and a, b. bentonite conc.) indicate no significant difference according to the Tukey test]

**Fig. 7:**
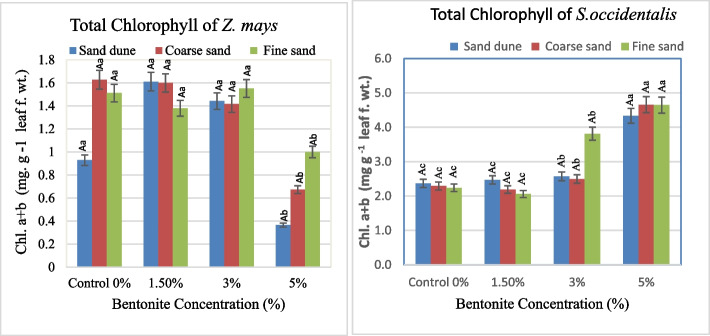
Total chlorophyll (*a* + *b)* contents of fresh leaves (mg. g.^−1^ fresh weight) of plants investigated at different bentonite concentrations (%) and soil textures [values having similar symbols (A, B. soil texture and a, b bentonite conc.) indicate no significant difference according to the Tukey test]

The effect of bentonite, soil texture and their interaction on growth and metabolic attributes in both *Zea mays* and *Senna occidentalis* plants was illustrated in Figs. 1, 2, 3, 4, 5, 6 and 7.

### Growth parameters

#### Length of organs

In Z*. mays* plants, the addition of bentonite (3–5% of soil weight) exhibited an increase in both shoot and root lengths (SL and RL). A maximum shoot length (42.3 cm) was observed in the calcareous (Cal.) soil. The coarse sandy soil (C. soil) encourages the root length and produces 24.0 cm (Fig. 1). The SL, RL (Fig. 1) and SL/RL ratio (Fig. 2) were greatly affected by 1.5% bentonite and produced the highest value (2.3) in fine sand soil (F soil). Similarly, in *S. occidentalis*, the applications of 3–5% bentonite stimulated both shoot and root length, particularly in plants grown in calcareous soil (Maximum length of shoot and root were 42.33 cm and 27.5 cm, respectively). The SL/RL ratio showed a similar pattern at different bentonite levels and soil textures (Fig. 2). Statistically, bentonite had a dominant role in the shoot length of *Z. mays* and root length of *S. occidentalis* as well as the SL/RL ratio of *Z. mays* (Table 1). The same role was played by the soil factor in the case of root length of *Z. mays* as well as SL and SL/RL ratio in *S. occidentalis.*

**Table 1:** F and share% values of the effect of soil texture, bentonite, and their interaction on the length (L) of the plant organs investigated

	**Organ parameter** **Source of variance**	**Shoot length (SL)**			**Root length (RL)**		**SL/RL Ratio**	
		**df**	**F**	**Share (%)**	**F**	**Share (%)**	**F**	**Share (%)**
***Z. mays***	Soil texture	2	2.05	9.08	7.90**	43.90	4.04*	13.24
	Bentonite	3	9.40**	62.36	1.45	12.07	5.56**	27.31
	Soil texture x Bentonite	6	2.15	28.56	2.64*	44.03	6.05**	59.45
***S. occidentalis***	Soil texture	2	10.46**	69.02	0.96	5.96	12.04**	56.58
	Bentonite	3	1.48	14.68	7.98**	74.01	2.78	19.63
	Soil texture x Bentonite	6	0.82	16.30	1.08	20.03	1.69	23.80

^*^Significant at 0.05 confidence level

^**^significant at 0.01 confidence level

#### Fresh weight

The fresh shoot weight (S Wt.) of *Z. mays* gained the highest value at 5% bentonite added to Cal. soil (Fig. 3). The same was true in the case of fresh root weight (R Wt.) without bentonite. It was found that, a maximum of S Wt. of 3.9 g and R Wt.

of 1.8 g. at 3–5% bentonite with substrate Cal. soil. The S Wt./R Wt. ratio of 3.6 existed when 5% bentonite in fine sandy soil. In *S. occidentalis*, the S Wt. and R Wt. obtained a high biomass at applied1.5% bentonite in plants grown in Cal. soil (1.6 g. and 1.2 g., respectively). The calcareous soil exerted a high S Wt./R Wt. ratio (3.6), while the lowest ratio was obtained in fine soil (Fig. 4). The F values and sharing % indicated that the bentonite effect was highly significant and had a dominant role on the R Wt. The same role of (Soil x bentonite) interaction was realized in the case of S Wt. (Table 2).

**Table 2:** F and share% values of the effect of soil texture, bentonite, and their interaction on the fresh weight (F Wt.) of the plant organs investigated

**Organ parameter** **Source of variance**		**Shoot weight (SWt.)**			**Root weight (RWt.)**		**S Wt./R Wt. Ratio**	
		**df**	**F**	**Share (%)**	**F**	**Share (%)**	**F**	**Share (%)**
***Z. mays***	Soil texture	2	0.57	7.80	0.81	17.19	0.99	12.87
	Bentonite	3	3.81*	77.87	0.43	13.86	1.39	27.19
	Soil texture x Bentonite	6	0.35	14.33	1.08	68.94	1.53	59.94
***S. occidentalis***	Soil texture	2	0.21	1.37	0.61	2.81	0.61	10.17
	Bentonite	3	2.84	28.39	9.16**	63.26	1.04	26.24
	Soil texture x Bentonite	6	3.51*	70.24	2.46	33.93	1.26	63.59

^*^Significant at 0.05 confidence level

^**^significant at 0.01 confidence level

#### Water content in shoot, root and soil

The presence of bentonite (3–5%) exhibited an increase in the water content of the shoot of *Z. mays* plants grown in various investigated soils and in the root at 3% bentonite (Fig. 5). In *S. occidentalis* the maximum content of shoot was shown at 5% bentonite in coarse sand and at 1.5% in Cal. soil, while high water content in root existed at 1.5% bentonite. The single factors and their interaction effect were significant (with some exceptions) on the water content organs of both species. Bentonite played a dominant role on the water content of shoot, whereas the soil texture had the same role in the case of the root (Table 3).

**Table 3:** F and share % values of the effect of soil texture, bentonite, and their interaction on the water contents of the soil and investigated plant organs

	**Water content**		**% Soil water content**		**% Plant water content of shoot**		**% Plant water content of root**	
	**Source of ** **Variance**	**df**	**F**	**Share (%)**	**F**	**Share (%)**	**F**	**Share (%)**
*Z. mays*	Soil texture	2	8.37**	49.28	12.85**	15.04	8.78**	48.42
	Bentonite	3	1.22	10.78	26.14**	45.92	4.97**	41.13
	Soil texture x Bentonite	6	2.26	39.93	11.11**	39.04	0.63	10.45
***S. Occidentalis***	Soil texture	2	12.40**	34.96	1.738	30.87	2.204	23.01
	Bentonite	3	3.95*	16.68	0.560	14.91	3.80*	59.49
	Soil texture x Bentonite	6	5.72**	48.35	1.018	54.22	0.559	17.50

*Significant at 0.05 confidence level

**significant at 0.01 confidence level

Applying bentonite exhibited an increase in the soil water, particularly in the calcareous sandy soil (Fig. 6). Bentonite, soil texture and / or their interaction had a significant effect on the soil content, where their interaction played the main role (Table 3).

Applying bentonite exhibited an increase in the soil water, particularly in the calcareous sandy soil (Fig. 6). Bentonite, soil texture and / or their interaction had a significant effect on the soil content, where their interaction played the main role (Table 3).

#### Chlorophyll content

Noticeably, the applied 1.5–3% bentonite stimulated the total chlorophyll content in *Z. mays* leaves which tended to a maximum (1.63 mg. g^−1^ fresh leaf weight) in calcareous and coarse sandy soils. Adversely, in *S. occidentalis,* the chlorophyll content was variable. Regardless of soil texture, the 5% bentonite concentration stimulates the total chlorophyll content in *S. occidentalis* (Fig. 7). Statistical analysis indicated that the effect of investigated factors was highly significant on the total chlorophyll of both species. Bentonite and interaction (soil texture x bentonite) had a dominant and subdominant role, respectively on the total chlorophyll of both investigated plants (Table 4).

**Table 4:** F and share % values of the effect of soil texture, bentonite, and their interaction on the total chlorophyll of the plant investigated

**Total chlorophyll** **Source of variance**		**Total Chl. of *Z. mays ***		**Total Chl. of *S. ****occidentalis *	
	df	F	Share (%)	F	Share (%)
Soil texture	2	5.16*	9.38	3.49*	1.67
Bentonite	3	26.59**	72.54	124.45**	89.17
Soil texture x Bentonite	6	3.31*	18.08	6.40**	9.17

^*^Significant at 0.05 confidence level

^**^significant at 0.01 confidence level

#### Metabolic compounds

**Fig. 8:**
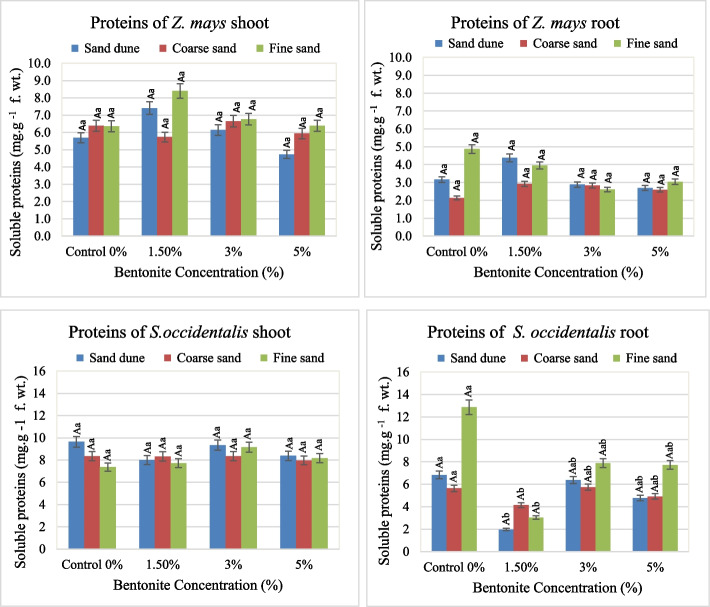
Total soluble protein contents (mg. g.^−1^ fresh weight) in shoots and roots of plants investigated at different bentonite concentrations (%) and soil texture [values having similar symbols (A, B. soil texture and a, b bentonite conc.) indicate no significant difference according to the Tukey test]

**Fig. 9:**
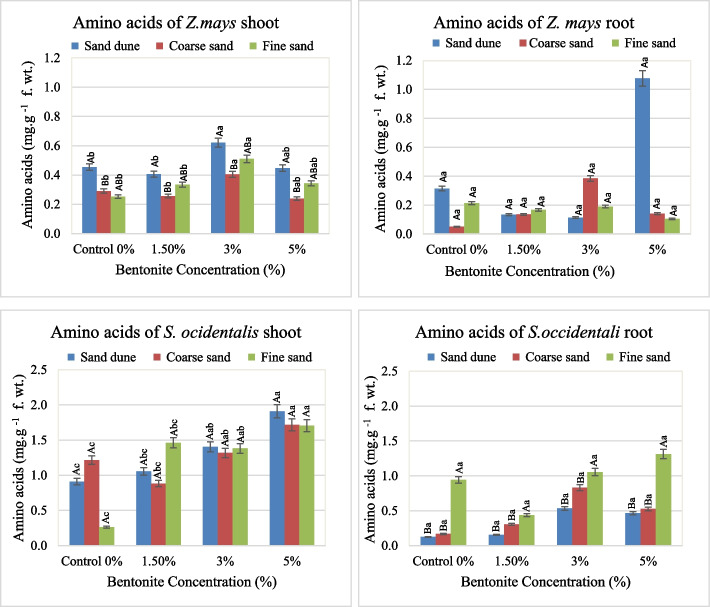
Free Amino acids contents (mg. g.^−1^ fresh weight) in shoots and roots of plants investigated at different bentonite concentrations (%) and soil textures [values having similar symbols (A, B. soil texture and a, b. bentonite conc.) indicate no significant difference according to the Tukey test]

**Fig. 10:**
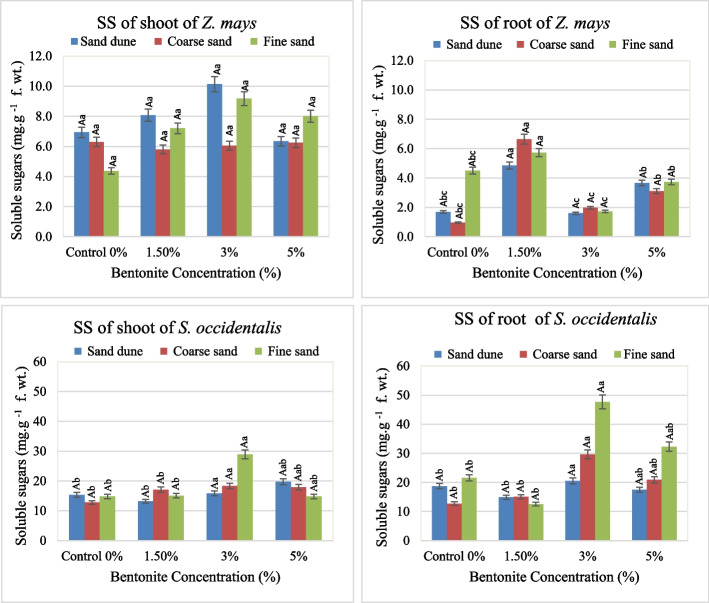
Total soluble sugar content (mg. g.^−1^ fresh weight) in shoots and roots of plants investigated at different bentonite concentrations (%) and soil textures [values having similar symbols (A, B. soil texture and a, b. bentonite conc.) indicate no significant difference according to the Tukey test]

Soil texture, bentonite and their interaction effect on the metabolic compounds. (Soluble proteins, free amino acids and soluble sugars) in the plants investigated were shown in Figs. 8, 9 and 10.

a **Total soluble proteins (S.P.)**
  In general, the soluble protein in the shoot showed a higher content than that in the root. The low concentration of bentonite (1.5%) had a boosting effect on the total soluble proteins in shoots and roots of *Z. mays*, particularly in fine soil (Fig. 8). Maximum soluble protein content was 8.4 and 4.4 mg g^−1^ fresh weight, respectively. The S.P. content in *S. occidentalis* was higher than that in the case of *Z. mays* plants. A maximum S.P. content was of 9.6 and 12.9 mg g^−1^ fresh weight in *Senna* shoot and root, respectively. While high S.P. content in the shoot was observed in calcareous soil, the same pattern in the root existed in the fine soil at different bentonite levels. Bentonite and soil texture singly had a highly significant effect on the soluble proteins of *S. occidentalis* root, where bentonite played the main role and soil texture role was subsidiary (Table 5).

**Table 5:** F and share % values of the effect of soil texture, bentonite, and their interaction on the total soluble proteins (S.P.) of the plant organs investigated

Content Source of variance	**df**	***Z. mays***					***S. occidentalis***					
		Shoot S.P.		Root S.P.			Shoot S.P.				Root S.P.	
		F	Share (%)	F	Share (%)	F			Share (%)		F	Share (%)
Soil texture	2	1.62	24.03	3.18	28.62			0.358		24.664	4.09*	24.845
Bentonite	3	1.74	38.74	2.17	29.21			0.296		30.621	5.83**	53.032
Soil texture x Bentonite	6	0.84	37.23	1.56	42.17			0.216		44.715	1.215	22.123

^*^Significant at 0.05 confidence level

^**^significant at 0.01 confidence level

b **Total free amino acids (A.A.)**
  Similarly, the low free amino acids were observed in root than those of shoot. In both shoot and root the applied bentonite (3–5%) stimulates the free amino acids content in *Z. mays* plants grown in calcareous soil. Also, calcareous and fine sandy soils exhibited an increase in the amino acids content in both organs of *S. occidentalis* with increases of bentonite concentration (Fig. 9).

The F values indicated that bentonite had a significant effect and played a major role in the free amino acids in different *Z. mays* and *S. occidentalis* organs. Exceptionally, in the case of *Senna* roots, the soil texture had the main role, and the bentonite role was subsidiary. Also, the interaction (soil texture x bentonite) had a dominant role on the A.A. of *Z. mays* roots (Table 6).

**Table 6:** F and share % values of the effect of soil texture, bentonite and their interaction on the total free amino acids (AA) of the plant organs investigated

Content Source of variance	df	***Z. mays***					***S. occidentalis***			
		Shoot A.A.			Root A.A.		Shoot A.A.		Root A.A.	
		**F**	**Share (%)**		**F**	**Share (%)**	**F**	**Share (%)**	**F**	**Share (%)**
Soil texture	2	6.94**		47.22	12.00**	17.90	0.449	1.287	10.35**	51.633
Bentonite	3	4.59*		46.90	8.20**	18.36	16.12**	69.216	4.77*	35.645
Soil texture x Bentonite	6	0.29		5.88	14.24**	63.75	3.43*	29.497	0.850	12.721

^*^Significant at 0.05 confidence level

^**^significant at 0.01 confidence level

iii. **c-Total soluble sugars (S.S.)**
  The addition of 3% bentonite had a boosting effect on the total soluble sugars of *Z. mays* shoots in calcareous soil. Likewise, in roots, the soluble sugar content (Maximum 6.6 mg, g leaf fresh wt.) was stimulating 1.5% bentonite in coarse soil. In fine sand, bentonite at 3% had a boosting effect on the soluble sugar content in both shoot and root of *S. occidentalis*. In general, the soluble sugar content in roots of both species investigated was higher than that of shoots (Fig. 10). The F value revealed that the bentonite or / and its interaction with soil texture had a significant effect and played the dominant and subdominant roles on the total soluble sugars in different tested plant organs except in the case of *Zea* shoots (Table 7).

**Table 7:** F and share % values of the effect of soil texture, bentonite and their interaction on the total soluble sugars (SS) of the investigated plant organs

Content Source of variance	df	***Z. mays***					***S. occidentalis***			
		Shoot S.S.			Root S.S.		Shoot S.S.		Root S.S.	
		**F**	**Share (%)**		**F**	**Share (%)**	**F**	**Share (%)**	**F**	**Share (%)**
Soil texture	2	2.74		23.27	5.53*	5.65	1.94	6.19	4.31*	22.94
Bentonite	3	2.91		37.11	49.38**	75.60	8.44**	40.46	6.48**	51.71
Soil texture x Bentonite	6	1.55		39.63	6.12**	18.75	5.56**	53.35	1.59	25.35

^*^Significant at 0.05 confidence level

^**^significant at 0.01 confidence level

#### Correlations between growth attributes, water content and metabolites

A significant positive correlation was found between the water content of soil, shoot and root with different growth parameters of *Z. mays* under the effect of soil texture x bentonite interaction (Table 8). Under the bentonite factor, the shoot length (SL) was positively correlated with free amino acids (A.A) and negatively with soluble proteins (S.P.).

**Table 8:** Significant correlations between the water content of soil, shoot and root with different growth parameters (length and weight) of *Z. mays* organs

Parameters Factors	Soil WC. x RL	Shoot WC. x S Wt	Root WC..x SL	Root WC. x SL/RL
Soil texture	0.997*	0.641	0.841	−1.00*
Bentonite	0.239	0.795	0.830	0.173
Soil texture x Bentonite	0.594*	0.665*	0.672*	−0.289

^*^Significant at 0.05 confidence level

^**^significant at 0.01 confidence level

Root weight (R Wt.) was negatively correlated with A.A. and total soluble sugars (S.S.) under the effect of soil texture and bentonite, respectively. While, S.S. had a positive correlation with shoot Wt./root Wt. ratio under soil texture factor (Table 9).

**Table 9:** Significant correlation coefficient between metabolites (S.P., A.A. and S.S.) and growth parameters (length and weight) of *Z. mays* organs

Content	Root S.S.		Root A.A.		Root S.P.
Parameter Factors	R. Wt	S Wt./R. Wt	SL	R. Wt	SL
Soil texture	−0.999*	0.999*	−0.268	0.661	−0.042
Bentonite	−0.004	−0.256	0.952*	−0.969*	−0.992**
Soil texture x Bentonite	−0.460	−0.310	0.371	−0.324	−0.277

^*^Significant at 0.05 confidence level

^**^significant at 0.01 confidence level

In *S. occidentalis*, the soil water content was positively correlated with root length and weight under the effect of interaction (soil texture x bentonite) and soil texture, respectively (Table 10). The effect of bentonite and its interaction with soil texture had a positive correlation between shoot S.P. and soil water. Root water content was negatively correlated with different metabolites under soil texture and its interaction with bentonite. Also, root weight was positively correlated with water content under the bentonite factor and its interaction with soil texture. Shoot length was positively and significantly correlated with shoot S.P. in soil texture treaments and with root A.A. in bentonite treatments.(Table 11).

**Table 10:** Significant correlation coefficient between soil water content with growth parameters (length and weight) and soluble proteins (S.P.) of *S. occidentalis* organs

% soil water content				
Parameters Factors	Root L	Shoot Wt.	Root Wt.	Shoot S.P.
Soil texture	0.978	0.524	1.000**	0.996
Bentonite	0.802	−0.954*	−0.714	0.984*
Soil texture x Bentonite	0.653*	−0.376	−0.351	0.761**

^*^Significant at 0.05 confidence level

^**^significant at 0.01 confidence level

**Table 11:** Significant correlation coefficient between root water content with root weight and metabolites (S.P., A.A. and S.S.) of *S. occidentalis* organs

%Root water content					
Parameters Factors	**Root S.P.**	**Root A.A.**	**Shoot S.S.**	**Root S.S.**	**Root Wt.**
Soil texture	−0.989	−1.000*	−1.000**	−0.999*	0.713
Bentonite	−0.873	−0.624	−0.543	−0.749	0.972*
Soil texture x Bentonite	−0.673*	−0.548	−0.463	−0.668*	0.667*

^*^Significant at 0.05 confidence level

^**^significant at 0.01 confidence level

## Discussion

Application of bentonite in different sandy soils of hot deserts has a potential role on the physical and chemical soil properties, ultimately on the plant health and productivity. The increase in root biomass and length enhances plant’s ability to absorb water from the soil depth [19]. Obtained data indicated that the addition of moderate amounts of bentonite (3%) to oolitic calcareous sandy soil produced a high length of both shoot and root systems of *Z. mays* and *S. occidentalis*. Likewise, the increased bentonite concentration in the same soil exhibited an increase in weight of both species' organs. While the shoot/root ratio of length and weight parameters exerted a maximum value at relatively high bentonite content (5% of dry soil Wt.) in fine sandy soil. This implied that the bentonite application in sandy soil promotes most morphological traits and water use efficiency in plants cultivated under water- deficit conditions [20]. Therefore, the presence of bentonite in drought- affected soils mitigates the negative impact of drought on the plant growth by improving soil moisture retention [21, 22]. Consequently, bentonite amendment facilitates the availability of water and nutrients for plant development [23]. Statistically, the bentonite had a dominant role on the length and weight of *Z. mays* shoot. This was true in the case of root length and weight of *S. occidentalis*. Whereas the soil texture played the same role on the shoot and root length of *Z. mays* and, *S. occidentalis*, respectively. Additionally, the soil texture X bentonite interaction substantially influenced the shoot / root length ratio in *Z. mays*. This means that the bentonite addition to growth substrates enhanced the agronomic attributes of experimental plants [24].

The water content in the aerial and sub-soil, a part of the plants investigated, was slightly affected by soil texture. The addition of moderate bentonite (3%) to the used sands, exhibiting an increase in the water content of shoot and root in both *Z. mays* and *S. occidentalis*. Apparently, bentonite accelerates the soil water retention, which helps the water uptake by plant roots and indicates a significant effect of bentonite or its interaction with soil texture on the soil water content, particularly in calcareous sands. The presence of colloidal soil particles also affects soil–plant interactions, nutrient quality, and water availability [25]. Therefore, adding soil supplements to sandy soil will improve soil accessible water consequently plant yield [26]. The F test and sharing % indicated that the bentonite played the main role and had a significant effect on the water content of *Z. mays* shoot and *S. occidentalis* root. Likewise, the same effect of soil texture and bentonite was recorded on the water content of *Z. mays* roots.

The effects of a soil amendment enhanced the sandy soil properties and plant photosynthesis activity resulting in more sustainable plant production. Hence, bentonite application enhanced photosynthetic activity [27]. At low bentonite levels (1.5–3.0%), total chlorophyll increased in *Z. mays* plants, whereas *S. occidentalis* showed a similar increase only at higher bentonite levels, particularly in fine sandy soil. Moreover, the highest chlorophyll content existed in the beds possessing the highest bentonite content [8]. The statistical analysis indicated that the bentonite had a highly significant role and the major role on the total chlorophyll of both investigated plants and the (soil texture x bentonite) had a secondary role. Accordingly, the presence of restricted amounts of bentonite in the growth substrates had a boosting effect on the chlorophyll contents in plants [24].

The accumulation of soluble osmolytes and proteins in plant tissues under water stress can provide a situation for the continuance of water uptake in the root zone. Therefore, amending soil with bentonite enhances the plant’s ability to absorb retained soil water. Within plants, the water bound partially depends on a colloidal property of soluble proteins. The water-soluble proteins investigated in plants responded to bentonite , with the strongest effects observed in fine sand. Accordingly, the soluble proteins in the roots of *Z. mays* and *S. occidentalis* exhibited an increase at moderate bentonite levels. The same existed in the shoot of *Z. mays* in fine sand and *S. occidentalis* in calcareous sand. Noticeably, the effect of bentonite played the main role on soluble proteins of *Z. mays* plants, and the soil texture had the secondary role.

The accumulation of osmotically active metabolites such as total soluble sugars and free amino acids in plants contribute to osmotic adjustment by lowering the internal osmotic potential, ultimately facilitating drought tolerance [28]. The data obtained elucidated that the free amino acids in both shoot and root of *Z. mays* and *S. occidentalis* had the highest value in the presence of 3–5% bentonite added to calcareous sandy soil. Moreover, the bentonite or its interaction with soil texture had a significant effect and dominant role on the free amino acids of various plant organs investigated. Generally, the bentonite played a crucial role (singly or in its interaction with sand) on the free amino acids of *Z. mays* and *S. occidentalis* cultivated in sandy soils, particularly calcareous sands. The positive effect of bentonite on the chemical composition and metabolites in plant tissues may be due to improving essential properties of sandy soil [10].

The soil supplied with moderate bentonite levels produced a maximum amount of total soluble sugars in the shoot and root of both *Z. mays* and S*. occidentalis* plants grown in fine sands. Therefore, the increased total soluble sugars of the root are accompanied by an increase in its capacity for water uptake and retention under drought conditions [29]. According to F values and sharing percentage % the total soluble sugars of the investigated plants were greatly affected by the bentonite factor, whereas the interaction (soil texture x bentonite) had a similar role on the S.S. of *S. occidentalis* shoot. This agreed with Bandian et al. [8] who reported that the soluble carbohydrate contents were enhanced by bentonite. The amending of soil with bentonite could significantly reduce the soluble carbohydrate levels accumulated under drought stress [27].

Several significant positive correlations were announced between the water content of the soil and plant organs with growth parameters of *Z. mays* under (bentonite x soil texture) interaction. Conversely, under the bentonite factor effect, the different metabolic compounds were negatively correlated with the growth parameters due to the translocation of metabolites to shoot and root construction. In agreement with Farghali and El-Aidrous [30], the total chlorophyll was positively correlated with soluble proteins and negatively correlated with free amino acids in root and participates in the construction of protein molecules in the shoot under the effect of bentonite, soil texture and / or their interaction. In the case of the *S. occidentalis* plant, the same correlations were found between different investigated parameters.

## Conclusion

Apparently, the obtained data revealed that the application of bentonite as a low- cost and abundant deposit in the Arabian deserts increases the nutrients availability and soil water retention. Results indicated that, the added 3% bentonite concentration to sandy soil, particularly calcareous sands improved the growth parameters of mesophytic plants such as length and biomass of shoots and roots. The total chlorophyll was markedly increased in plants due to high nutrient availability. Adding bentonite to the sand substrate resulted in the highest amounts of water-soluble metabolites in the plants. Comparatively, the cultivated *Z. mays* exhibited superior growth parameters compared to the wild *S. occidentalis.* Otherwise, the wild mesophyte outperforms the cultivated plant in the metabolic compound contents. This reflected an advantageous strategy of wild plants for an increase in osmo-metabolic adjustment which is helpful in the drought tolerance of plants exposed to arid and semi- arid conditions.

## Data Availability

All the data are in the published article.

## Abbreviations

*Z. mays*: *Zea mays*
*S. occidentalis*: *Senna occidentalis*
Cal. Soil: Oolitic calcareous sandy soil in Mediterranean coastal dunes
C. soil: coarse sandy soil in Wadi El- Assiuti and the eastern desert
F. soil: fine sandy soil in Kharga Oasis region in the western desert
S Wt.: shoot weight
R Wt.: root weight
S Wt./R Wt. ratio: shoot weight/ root weight ratio
S.L: shoot length
R.L: root length
S.L./R.L: shoot length/ root length ratio
Soil x bentonite: interaction between soil and bentonite
S.P.: soluble proteins
A.A.: amino acids
S.S.: soluble sugars
